# CAREx: context-aware read extension of paired-end sequencing data

**DOI:** 10.1186/s12859-024-05802-w

**Published:** 2024-05-10

**Authors:** Felix Kallenborn, Bertil Schmidt

**Affiliations:** Department of Computer Science, Johannes Gutenberg University Mainz, Mainz, Germany

**Keywords:** Next-generation sequencing, Pseudo-long reads, GPU

## Abstract

**Background:**

Commonly used next generation sequencing machines typically produce large amounts of short reads of a few hundred base-pairs in length. However, many downstream applications would generally benefit from longer reads.

**Results:**

We present CAREx—an algorithm for the generation of pseudo-long reads from paired-end short-read Illumina data based on the concept of repeatedly computing multiple-sequence-alignments to extend a read until its partner is found. Our performance evaluation on both simulated data and real data shows that CAREx is able to connect significantly more read pairs (up to $99\%$ for simulated data) and to produce more error-free pseudo-long reads than previous approaches. When used prior to assembly it can achieve superior de novo assembly results. Furthermore, the GPU-accelerated version of CAREx exhibits the fastest execution times among all tested tools.

**Conclusion:**

CAREx is a new MSA-based algorithm and software for producing pseudo-long reads from paired-end short read data. It outperforms other state-of-the-art programs in terms of (i) percentage of connected read pairs, (ii) reduction of error rates of filled gaps, (iii) runtime, and (iv) downstream analysis using de novo assembly. CAREx is open-source software written in C++ (CPU version) and in CUDA/C++ (GPU version). It is licensed under GPLv3 and can be downloaded at (https://github.com/fkallen/CAREx).

**Supplementary Information:**

The online version contains supplementary material available at 10.1186/s12859-024-05802-w.

## Background

Next generation sequencing (NGS) platforms such as Illumina typically produce reads of a few hundred base pairs in length with high coverage at low cost. Downstream analysis of NGS data, however, often benefits from longer reads since repeat structures could more easily be resolved.

Although third-generation sequencing technologies can generate significantly longer reads of more than 10.000 bp, Illumina platforms are still frequently used in practice [1].

NGS technologies often use paired-end (PE) sequencing, where pairs of short reads are produced from two ends of a DNA fragment. The average distance between the far end of reads is called the *insert size*. Given such a read pair, the redundancy (high coverage) of the considered NGS data could be used to reconstruct the missing nucleotides in-between the reads of the pair. This results in elongated reads, so called *pseudo-long reads*. It has been shown that the construction of these artificially long reads from real short reads can be beneficial for improving de novo genome assembly [2], metagenomics [3], and variant detection pipelines [4].

Early work addressed only the simple case, where the insert size is less than the sum of read lengths of the pair. In this case both reads overlap. A pseudo-long read can then be obtained by finding the best overlap with respect to the insert size. This approach is used by FLASH [5], COPE [6], and PEAR [7].

Extending short read pairs to pseudo-long reads is more challenging if the insert size exceeds the sum of read lengths. In this case, there are unknown nucleotides which need to be determined from other overlapping reads in order to produce a pseudo-long read. This problem can be formulated as a small local assembly operation, creating a small contig that is delimited by the two reads of a pair.

Existing approaches which extend a given read until its partner (mate) is found are based on detecting overlaps between *k*-mers. They can be distinguished by their utilized data structures and specific extension rules. GapFiller [8] and Eloper [9] employ simple seed-and-extend strategies based exact matches using hash tables. Konnector2 [4] constructs a deBruijn graph from the *k*-mers of all reads. Subsequently, the deBruijn graph is traversed for each read pair to find a connecting path, which is then translated into a pseudo-long read. MaSuRCA [10] is a genome assembler that is build around the construction of super-reads. It extends a read on each end base per base as long as there is only exactly one possible base to append. The *k*-mer spectrum of the input read dataset is used to verify that only one possibility exists. Aside from contig assembly, the construction of pseudo-long sequences can also be used by genome assemblers to bridge gaps between contigs within a scaffold [11–13]. PLR-GEN [3] creates pseudo-long reads from clustered metagenomic short reads based on given reference genome sequences.

Although these previous approaches can be computationally efficient, they might fail to detect correct extensions in scenarios where exact matching based on *k*-mers is insufficient.

We present CAREx—a new read extension algorithm for Illumina PE data based on indel-free multiple-sequence-alignment (MSA). The key idea is to build MSAs of reads sequenced from the same genomic region. Extension is performed based on consensus MSA columns. While our approach aims to connect the reads of a read pair, limited extension in outward direction, i.e. extension at the $5'$ end, can also be performed. CAREx is inspired by our recent work on sequencing read error correction [14, 15]. MSA-based approaches have also been shown to be effective for long read self-correction and assembly polishing [16]. Although, such methods can be computationally complex, we gain efficiency by applying a variant of minhashing to quickly find a set of candidate reads which are similar to a query read with high probability and aligning with fast bit-parallel algorithms.

We demonstrate that our approach is beneficial to the

- percentage of read pairs connected to pseudo-long reads,
- reduction of error rates of filled gaps,
- runtime, and
- downstream analysis using de novo genome assembly.

The number of perfectly connected read pairs of CAREx exceeds the total number of connected read pairs of other tools. In general, we are able to produce significantly more connected read pairs on both simulated data and real-world data.

For example, CAREx and Konnector2 produce 181M and 107M error-free connections, respectively, on a real-world human dataset.

Our GPU-accelerated version of CAREx is up to 9.5 times faster than CPU-based Konnector2 on the used datasets. Last, we show that de novo genome assembly of reads connected with CAREx yields contigs with fewer misassemblies than those connected by Konnector2.

## Implementation

### Algorithmic approach

We perform targeted local assembly to produce contigs which are delimited by the two reads of a read pair. This is achieved by repeatedly constructing MSAs centered around the $3'$ end of the currently computed contig. The consensus sequence of an MSA is used to elongate the contig.

MSAs are constructed from reads of the input NGS dataset, adapting the steps from the well known center star alignment approximation algorithm [17]. Reads are aligned to a center sequence, which is the contig. The computed alignments are subsequently arranged into an MSA. We target Illumina reads where the dominant sources of errors are substitutions. Thus, our alignments do not consider indels. Alignments are efficiently computed using a bit-parallel hamming distance algorithm for each possible overlap between center sequence and read.

A key challenge of this approach is to quickly identify reliable reads which are similar to the $3'$ end of the contig. Hash tables are utilized for this purpose. Minhashing is a specific locality sensitive hashing subsampling technique that was originally introduced by search engines to detect near duplicate web pages [18]. In recent years, it has proven beneficial for the processing of NGS data in the context of genome assembly [19], metagenomics [20, 21], read mapping [22], and error correction [15].

We apply the concept of minhashing to compute multiple *k*-mer hashes per read. Let $F=(f_1,\ldots , f_h)$ be a collection of *h* hash functions, and $T=(t_1,\ldots ,t_h)$ a collection of hash tables. The following operations are performed per read to construct a database of *k*-mer hashes. Each hash function is applied to each *k*-mer of read $r_i$ to produce *k*-mer hashes. Let $m_x$ be the smallest observed hash value for hash function $f_x$. Then the so called minhash signature $S=(m_1,\ldots ,m_h)$ is given by the set of all smallest hash values. Subsequently, the key-value pair $(m_x, i)$ is inserted into hash table $t_x$, $\forall x$. After hash tables are constructed, the minhash signature of any sequence, for example a substring of a contig, can be queried to obtain read IDs of reads which are likely to share a *k*-mer with that sequence.

### Workflow

Our algorithm consists of two separate phases:

1. database construction,
2. read extension.

During database construction the input FASTQ/A files are parsed and reads are stored in memory. Sequences are converted into a 2-bit format. Ambiguous nucleotides are deterministically replaced by A,C,G, or T. For quality scores, we offer an optional lossy compression to reduce memory usage. Subsequently, hash tables are constructed following the steps explained above.

**Fig. 1:**
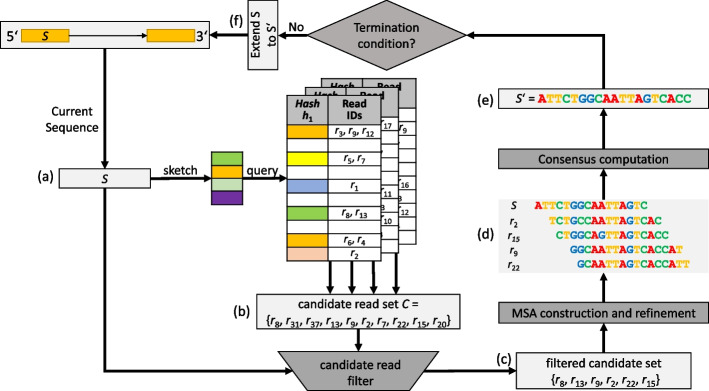
Workflow of a primary extension task for a given read pair: **a** The current sequence *S* is extracted from the $5'$ end. **b** The sketch of *S* is determined by minhashing and used to query the hash tables. The retrieved reads form the candidate read set *C*. **c** All reads in *C* are aligned to *S*. Reads with a relatively low pairwise alignment quality are removed, resulting in the filtered candidate reads. **d** An initial MSA is constructed and refined by removing candidate reads with a significantly different pattern from *S*. **e** The consensus string is computed leading to an extended sequence $S'$. **f** Depending on the termination conditions (see text) the iteration proceeds using the extended sequence $S'$ at the $5'$ end

Computation of pseudo-long reads in CAREx revolves around the processing of so called extension tasks. Consider a starting sequence *S*. An extension task iteratively appends suitable nucleotides to the $3'$ end until a condition is met. This is performed by constructing MSAs of reads similar to *S*. Then, a substring of the MSA consensus is used to elongate *S*. The workflow is illustrated in Fig. 1.

**Fig. 2:**
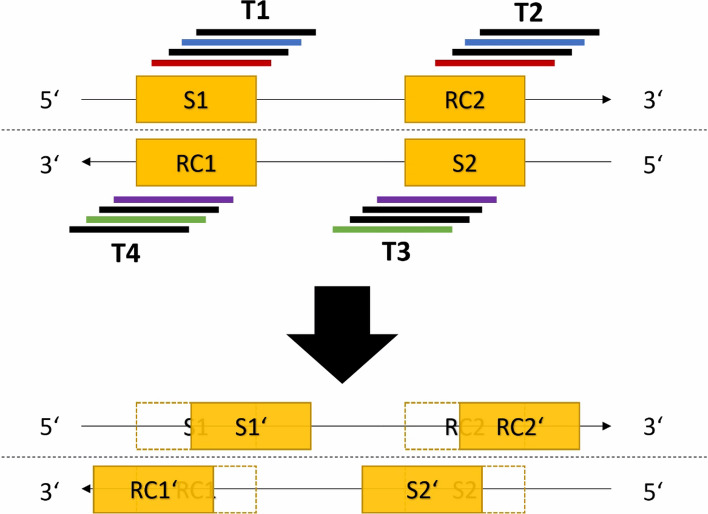
Layout of the four starting sequences for extension tasks. T1: starting sequence *S*1, T2: starting sequence *RC*2, T3: starting sequence *S*2, T4: starting sequence *RC*1

Consider two DNA sequences *S*1 and *S*2 that form a read pair. *RC*1 and *RC*2 denote their respective reverse complement sequences. For this read pair, four extension tasks (T1, T2, T3, T4) are created with different starting sequences as shown in Fig. 2.

T1 and T2 are considered partner tasks, as well as T3 and T4. T1 and T3 are primary tasks with the goal of finding the end of a pseudo-long read, i.e. T1 (T3) will stop if *RC*2 (*RC*1) has been reached. T2 and T4 are auxiliary tasks connected to tasks T1 and T3, respectively. Their main purpose is to identify reliable pairs of candidate reads. T2 (T4) stops as soon task T1 (T3) stops. In addition, each of the four tasks can end if it is no longer possible to perform an extension of the sequence. The tasks are processed simultaneously. After all tasks are completed, a pseudo-long read of the read pair is constructed, if possible, from the generated extended sequences of T1 and T3. In addition, if outward extension is requested, the sequences produced by T2 and T4 can be appended to the result at the appropriate ends.

In the following we explain the individual steps performed in a single processing iteration of an extension task.

- The minhash sketch of the task’s current sequence *S* is computed and subsequently queried against the database. This results in a set *C* of query results consisting of read IDs (candidate read set).
- Candidate read sequences are fetched from memory, and are aligned to *S* via multiple fast hamming distance calculations. Only alignments with $50\%$ overlap and at most 5 mismatches are considered. Additionally, since we aim to extend *S* on the $3'$ end, only alignments are computed where the first letter of a candidate is included in the alignment overlap, i.e. the candidate cannot be positioned left of *S*.
- Candidate reads are filtered by their alignment quality. Our filter can use the information about paired reads to simultaneously apply a filter to the candidate reads, and the candidate reads of the partner task. If the mate of a candidate read is present in the candidate list of the partner task, both candidate reads are kept unconditionally. Unpaired candidates are removed depending on their alignment overlap. Let $O_i = overlap / |S|$ denote the relative overlap size between aligned unpaired candidate *i* and *S* and $T = \max _i(\lfloor O_i * 10 \rfloor / 10)$. Then unpaired candidates with $O_i < T$ are removed. In case the partner task has terminated, all candidates are treated as unpaired.
- The remaining, filtered candidates are arranged into an initial MSA *M* centered around *S*. *M* is further refined by inspecting its column contents to remove candidates which may originate from inexact repeat regions. Those candidates may lead to columns with unclear consensus nucleotides. Specifically, the filter identifies columns that are covered by *S* where a non-consensus nucleotide *x* of this column occurs in at least $0.3 \times coverage$ of the rows for that specific column. If *S* has the same nucleotide *x* in this column, all candidate reads without *x* in this column are removed. Otherwise, those candidates with *x* are removed.
- The consensus string of the refined MSA $M_r$ is used to compute an extended sequence $S'$ from *S*. Let *i* be the first column of $M_r$ that is not part of the anchor read *S*. The algorithm determines column $j > i$ where $j-i \le stepsize$, the coverage of column $j \ge m$, and *j* maximal. Using $n = j+1-i$ the extended sequence $S'$ is computed as $S' = S[n:|S|] + consensus[i:j+1]$; i.e., $S'$ is obtained by appending the consensus substring of length *n* to *S* and removing the first *n* nucleotides from *S*. *stepsize* and *m* are parameters with default values of $stepsize = 20$, and $m=3$.

There are three different outcomes of the computation of $S'$.

1. The algorithm may fail to compute $S'$ if either column *i* or column *j* do not exist. This can be the case if the number of columns in $M_r$ is |*S*|, or if all columns to the right of column *i* have low coverage. If computation fails, the task terminates.
2. When computing $S'$ in a primary task the algorithm may find that the target sequence, i.e. the read’s mate, has been reached. In that case, the task terminates, as well.
3. Extension succeeds without reaching the mate. The task’s sequence *S* is updated to $S'$ and the next task iteration begins

After all four tasks of a read pair are finished, a post-processing step merges the constructed pseudo-long reads of each task.

The construction of the final extended read from the four tasks can be controlled by a parameter called *strict mode*. We currently provide three different types of strictness.

Strict mode 2 is the most restrictive. If both T1 and T3 have finished after finding the target sequence, and both tasks produced a connection of same length between the reads, this connection is used if the hamming distance between the connections of the two tasks is less than some parameter *x*. By default, we require an exact match. In all other cases, the read pair remains unconnected.

Strict mode 1 includes mode 2. In addition, mode 1 can handle the case when only one of T1 or T3 found the mate. Assume T1 found its mate and the size of the filled gap is *s*. Then the connection is used if the overlap between the filled gap of *T*1 and the incomplete filled gap of T3 is at least of size *y* (default: $50\%$ of *s*), and the overlap contains at least $z\%$ matches (default: $95\%$ of *s*).

Strict mode 0 is the least restrictive. If either T1 or T3 have finished after finding the target sequence, their corresponding result sequence is used as the pseudo-long read. For some read pairs, neither of both primary tasks may have finished successfully. Yet, they may have computed sufficiently long, but incomplete, pseudo-long reads. If agreeable with the estimated insert size, the result sequences of both tasks are merged to form the pseudo-long read of that read pair. Otherwise, the read pair is not connected. The merge will only be performed if the suffix of one sequence can be overlapped with the prefix of the reverse complement of the second sequence by at least 40 positions with at most $5\%$ mismatches. In case of multiple possible overlaps, the longest overlap is chosen.

For all levels of strictness, if it was possible to connect *S*1 and *S*2, results of T2 and T4 may be used to further elongate the pseudo-long read on both ends. It is achieved by simply appending the extended nucleotides of those tasks to the appropriate ends of the pseudo-long read, respecting the correct strand.

In all cases, for positions in the pseudo-long reads which correspond to the original reads the original nucleotides are used, i.e. no modifications to the original reads are performed.

### Parallelization

CAREx employs two different parallelization strategies to enable fast computation on modern workstations. Our C++ implementation targets CPU workstations using C++ threads for multi-threading. The core algorithm is trivially parallelizable because different read pairs can be processed independently.

In addition, we provide a CUDA implementation targeting GPUs. CUDA-capable GPUs use a many-core architecture that can process thousands of threads in parallel. For best utilization, multiple batches of read pairs are extended simultaneously. GPUs allow for different parallelization schemes. For example, all alignments of candidate reads to their respective sequence *S* are computed in parallel using a single GPU thread per alignment. In contrast, MSAs are constructed in cooperative fashion where multiple GPU threads per MSA are used to update multiple columns at the same time.

For best performance, hash tables can be stored in GPU memory. However, they require a significant amount of memory and GPU memory can be scarce. Since GPU memory can be a limiting factor hash tables can be placed in either CPU memory or GPU memory. Our GPU hash tables are based on the Warpcore library [23].

If the hash tables are not placed on the GPU, read signatures are calculated on the GPU and are subsequently transferred to the CPU for hash table queries. Query results then need to be copied back to the GPU. CPU-side hash table queries are significantly slower than GPU-side hash table queries and are a performance bottleneck. To improve the performance with CPU-side hash tables, we use two sets of threads of different size which can communicate via queues. The first set of threads is responsible for hash table accesses on the CPU whereas the second set of threads performs read extension using the GPU. After each extension iteration the batch is transferred to the hasher threads and read extension resumes using a (possibly) different batch for which hash table accesses have already completed. Depending on the actual hardware, one or two threads which perform extension are sufficient, but need to be complemented with 8 or more threads responsible for (slow) hash table lookups.

## Results

To assess performance, we present results based on both simulated and real Illumina datasets. We used standalone tools that can produce one pseudo-long read per input read pair. We did not consider the build-in gap closing functionality of assemblers since they only operate at contig-level. Thus, the results of CAREx are compared to GapFiller 2.1.2 and Konnector 2.2.4. MaSuRCA and Eloper are not used because they do not output individual pseudo-long reads per read pair. Additionally, Eloper did not finish on the used datasets. Furthermore, PLR-GEN is only applicable to metagenomic data with provided reference genomes while the other tested tools are reference-free.

For both types of datasets, the number of connected read pairs is determined, as well as the accuracy of extensions. In addition, the quality of de novo assembly using extended real-world reads is investigated.

Simulated datasets were generated using the ART read simulator [24]. We have used three different reference genomes: *Drosophila melanogaster (D. melanogaster)*, *Caenorhabditis elegans (C. elegans)*, and *Human Chromosome 14*. Most of our generated datasets have a coverage 30x or 60x and read length 100, with insert sizes (stddev) of: 300(5), 500(10), 1000(30). We also include a simulated dataset with read length 150 and insert size 500(150). Additionally, we include a simulated mate-pair dataset with a significantly larger insert size (2500) compared to the other datasets. Note that read lengths *l* of a read pair are included in the specified insert size; i.e., the average gap size between the two reads of a read pair is insert size $- 2\cdot l$. Table 1 lists the datasets.

**Table 1:** Simulated (S1–S8) and real (R1–R4) paired-end datasets used for evaluation

Name	Organism	Cov.	Read pairs (M)	Length	Insert size	SD
S1	*C. elegans*	30x	15.0	100	300	5
S2	*C. elegans*	30x	15.0	100	500	10
S3	*C. elegans*	30x	15.0	100	1000	30
S4	*C. elegans*	60x	30.1	100	1000	30
S5	*D. melanogaster*	30x	18.0	100	500	10
S6	*Human* Chr. 14	30x	13.2	100	500	10
S7	*C. elegans*	30x	10.1	150	500	150
S8	*C. elegans*	30x	10.1	150	2500	100
R1	*D. melanogaster*	64x	37.9	101	598	39
R2	*Human* Chr. 21	33x	6.7	100	312	14
R3	*Human* (NA12878)	30x	304.6	148	546	117
R4	*Human* (NA24385)	31x	311.8	148	568	159

S8 is a mate-pair dataset

All tools provide different settings. The exact list of program arguments can be found in Additional file 1: Section A. For GapFiller we specified insert size and standard deviation. Konnector2 was run with *k*-mer size 32. The size of the Bloom filter was set such that the reported false-positive rate is around $0.3\%$. CAREx used 48 hash tables with *k*-mer size 20. For both Konnector2 and CAREx we set the maximum allowed pseudo-read length to $insert size + 4 \times stddev$, and the minimum length to $\max (2\cdot readlength, insert size - 4 \times stddev)$. Thus, we do not consider the simple case of overlapping reads.

Real-world datasets come from *D. melanogaster* (SRR988075), *Human* Chromosome 21 (NA19240 Illumina Data Library), and full *Human* (NA12878, NA24385) and are also listed in Table 1. For R3 we concatenated the data of SRR2052337, SRR2052338, SRR2052339, SRR2052342, SRR2052348, SRR2052352, and SRR2052354. For R4 we concatenated the data of SRR1766553, SRR1766558, SRR1766560, SRR1766562, SRR1766581, SRR1766585, and SRR1766588.

### Extension of simulated reads

Since the location of each simulated read in its reference genome is known, detailed statistics of the generated pseudo-long reads are possible. This allows us to compute the edit-distance in the filled gap. We evaluate read extension by the number of connected reads pairs, as well as their error rate within the gap filled between the reads. Error rate is computed as the sum of edits over the sum of filled gap lengths. The edit-distance is computed by comparing the filled gap to the corresponding positions in the reference genome.

**Fig. 3:**
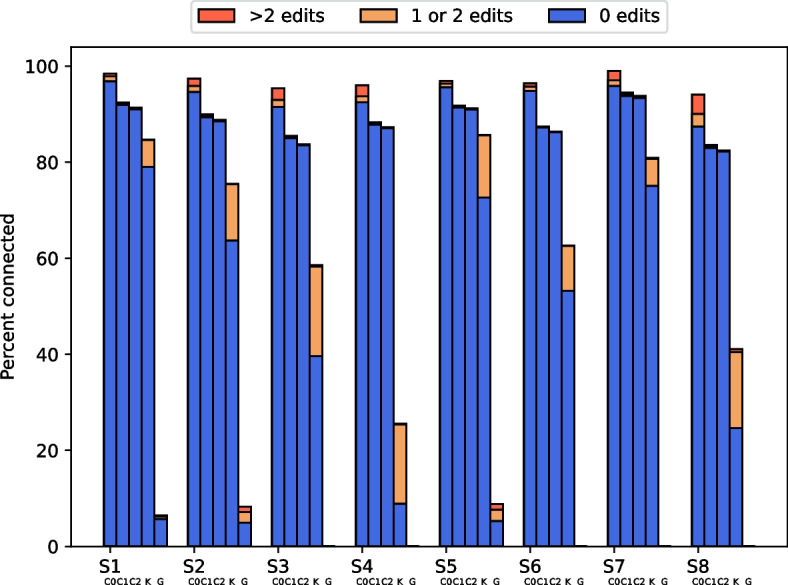
Percentage of connected read pairs on simulated data and their error numbers. C0: CAREx strict 0, C1: CAREx strict 1, C2: CAREx strict 2, K: Konnector2, G: GapFiller. GapFiller did not finish (DNF) for S3, S4, S6, S7, S8

Figure 3 shows the percentage of connected read pairs per dataset and the percentage of error-free connected read pairs for all three tools. The size of the Bloom filter was set to 10 GB for Konnector2 for all simulated datasets which yields an average reported false-positive rate of around $0.27\%$.

CAREx achieves a high percentage of connected read pairs and error-free connected read pairs across all simulated datasets, with up to $99\%$ connections for dataset S7 with strictness 0. As expected, using a more conservative mode (strict mode 1 or 2) decreases the number of successful connections by around $10\%$ of all pairs. CAREx is able to produce significantly more pseudo-long reads than Konnector2 and GapFiller. With only around $6\%$ of reads connected, GapFiller consistently performs worst. Increasing the dataset coverage to 60x seems to be beneficial for CAREx. Both the percentage of connected read pairs and percentage of error-free extensions increases.

**Table 2:** Total error rate of filled gaps

Dataset	CAREx			Konnector2	GapFiller
	Strict 0	Strict 1	Strict 2		
S1	0.00059	0.00013	0.00006	0.00078	0.00278
S2	0.00081	0.00012	0.00005	0.00063	0.00396
S3	0.00128	0.00009	0.00003	0.00070	DNF
S4	0.00127	0.00010	0.00003	0.00111	DNF
S5	0.00026	0.00006	0.00004	0.00056	0.00371
S6	0.00030	0.00004	0.00002	0.00062	DNF
S7	0.01072	0.00252	0.00151	0.00216	DNF
S8	0.00268	0.00012	0.00005	0.00122	DNF

Table 2 lists the total rate within the filled gap for all tools. Konnector2 and CAREx (strict 0) have a similar error-rate on S1-S6,S8, whereas Konnector2 produces much less edits on dataset S7. With strict mode set to 1, error-rate of CAREx drops by up to one order-of-magnitude, and further decreases by a factor of 2 on average with strict mode 2.

The computation of edit-distance measures the combined effect of two sources of errors in the filled gap. First, an extension algorithm may select the wrong nucleotide to append and thus introduces a substitution error. Second, the algorithm may produce a pseudo-long read of incorrect total length which leads to indels compared to the reference genome region. To be able to find our main contributor to errors, substitutions or indels, we computed a second set of error-rates that use a modified edit-distance score. The modified edit-distance is computed by subtracting the absolute length difference between expected gap size and produced gap size from the original edit-distance.

**Table 3:** Total error rate of filled gaps excluding length differences

Dataset	CAREx			Konnector2	GapFiller
	Strict 0	Strict 1	Strict 2		
S1	0.00035	0.00009	0.00004	0.00071	0.00270
S2	0.00041	0.00006	0.00003	0.00053	0.00385
S3	0.00045	0.00003	0.00001	0.00041	DNF
S4	0.00042	0.00003	0.00001	0.00082	DNF
S5	0.00015	0.00004	0.00002	0.00051	0.00365
S6	0.00020	0.00002	0.00001	0.00050	DNF
S7	0.00029	0.00005	0.00003	0.00032	DNF
S8	0.00045	0.00002	0.00001	0.00018	DNF

Table 3 lists the corresponding error rates for the modified edit-distance. Compared to the original edit-distance, the error-rates are reduced and have less variation across the different datasets. For dataset S7, modified error-rates for CAREx are up to fifty times smaller, indicating significant contribution to the error-rate by length differences. This large difference can be explained by the standard deviation of insert size which affects the target pseudo-long read length. For S7, a target length between 300 and 1100 was specified. CAREx terminates extension as soon as the mate can be placed at the end of the pseudo-long read within that range with a small hamming distance. However, this leads to issues with (inexact) repeat regions that allow multiple target positions for the mate, especially if those positions are far apart. We always choose the first position. Thus, if the length is incorrect, the produced pseudo-long read is more likely too short rather than too long. If the insert size was known precisely, this would not be an issue.

**Table 4:** Total error rate of filled gaps of dataset S7

	All	A	B
*CAREx strict 0*			
Pairs connected	10,016,848	80,296	9,936,552
Error-free pairs	9,695,451	5127	9,690,324
Error-rate	0.01072	2.05549	0.00397
*CAREx strict 1*			
Pairs connected	9,558,861	20,232	9,538,629
Error-free pairs	9,488,850	372	9,488,478
Error-rate	0.00250	4.5121	0.00050
*CAREx strict 2*			
Pairs connected	9,487,590	12,348	9,475,242
Error-free pairs	9,442,647	317	9,442,330
Error-rate	0.00151	4.05969	0.00028
*Konnector2*			
Pairs connected	8,185,781	3840	8,181,941
Error-free pairs	7,595,997	375	7,595,622
Error-rate	0.00216	1.46986	0.00180

Category A contains all read pairs with multiple potential mate positions. Category B contains the remaining read pairs

To provide more insights into this source of length errors, we performed an additional evaluation of the results for dataset S7. From the set of extended read pairs we used the known locations within the reference genome to identify those pairs where any of the two reads could be placed at multiple positions in the target range, subject to a maximum hamming distance of 9. We separated those read pairs and re-evaluated the error-rate of the resulting two sets of read pairs using the standard edit-distance. The results are presented in Table 4. For CAREx it shows that the number of read pairs with such ambiguous mate locations is a tiny fraction of the total connected pairs. However, their error-rate is up to four orders-of-magnitude greater than those of the remaining pairs, thus having a significant contribution to the reported overall error-rate. For Konnector2, the gain in accuracy is less prominent that in CAREx meaning that there are substantial contributions by other types of read pairs. When comparing Tables 3 and 4 it can be noticed that although leaving out those pairs with ambiguous mate locations greatly increases accuracy (Column B), not considering any length errors still yields a one order-of-magnitude better accuracy. This indicates the presence of different sources of length errors. For example, there could be a repeat in the genome section preceding the mate. CAREx does not perform any special handling of repeats, and may or may not recognize or resolve this repeat correctly.

Last, we have briefly evaluated the error rate and pseudo-long read lengths of outward extensions of CAREx. The following values are averaged over datasets S1-S7 for strict mode 0. The average pseudo-long read length is $1.9 \times (insert size + 4 \times stddev)$, with a maximum length of $2.7 \times (insert size + 4 \times stddev)$. The error rate of nucleotides extended in outward direction is 3.5 times higher than the error rate within the filled gap.

Overall, CAREx produces the best results on these datasets; i.e., it produces the most pseudo-long reads, as well as the most error-free pseudo-long reads. Exact numbers of error-free connections per program and dataset are listed in Additional file 1: Section B.

### Extension of real datasets

For our real-world evaluation we chose a similar approach to simulated reads in terms of error-rate comparison. In addition, we performed de novo assembly of extended reads of Konnector2 and CAREx.

For the real-word datasets no reference positions are known. To be able to determine the error rate in the gap, extended reads were aligned to a reference genome using BWA 0.7.17 [25]. Alignments were filtered to remove secondary and supplementary alignments which results in at most one alignment per read. In addition, clipped alignments are removed, as well. Positions of the filled gaps were then extracted from the alignments and compared to the reference genome to compute the edit-distance. Note that since only edits within the filled gaps are considered, the total edit-distance already computed by BWA cannot be used.

The Bloom filter size for Konnector2 was set to produce a false-positive rate similar to the simulated reads. Specifically, it was set to 15*G* and 3*G* for R1 and R2, respectively. We used a size of 200*G* for R3 and R4. This results in a reported false-positive rate of $0.26\%$, $0.29\%$, $0.31\%$, and $0.31\%$, respectively. For datasets R3 and R4, we set the minimum / maximum insert size to 300 / 1100. For R1 and R2 we follow the previously stated formula for target pseudo-read length. To be able to fit the whole dataset in GPU memory, CAREx employed a lossy compression of the quality scores on dataset R3 and R4, using 2 bits instead of 8 bits per letter.

Recall that in CAREx original read positions in the pseudo-reads are left unmodified. Konnector2, however, performs error-correction on these positions by default. These differences could impact the mapping process and the assembly when read pairs from Konnector2 and CAREx are processed differently solely based on original read positions, despite having the exact same filled gap. Thus, for our real-world evaluation we performed additional extensions with Konnector2 with disabled built-in error-correction via program argument –preserve-reads.

**Fig. 4:**
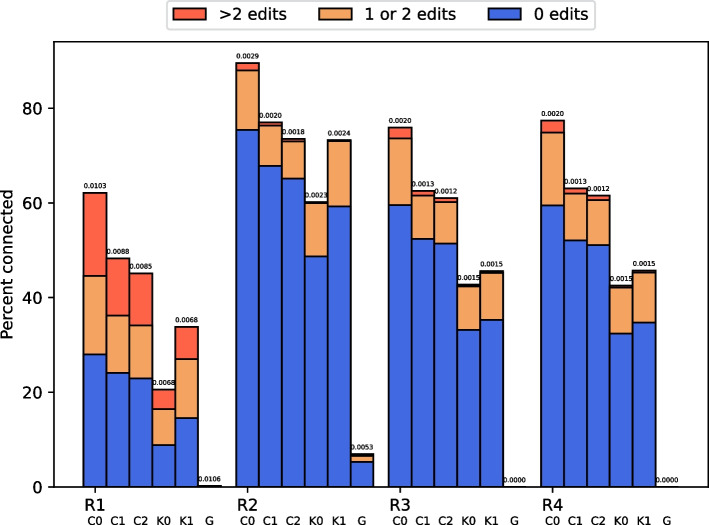
Percentage of connected read pairs on real-world datasets. The total error-rate is displayed at the top. C0: CAREx strict 0, C1: CAREx strict 1, C2: CAREx strict 2, K0: Konnector2 with preserve-reads option, K1: Konnector2 without preserve-reads option, G: GapFiller

Figure 4 shows the percentage of connected read pairs and the corresponding fraction of edit-free filled gaps, as well as the fraction of filled gaps with edit-distance $\le 2$. Addtionally, the error-rate is reported, as well. Neither CAREx nor Konnector2 were run with enabled outward extension. CAREx produces the most connected read pairs and the highest amount of error-free connections on all three datasets. In terms of error-rate, Konnector2 is better than CAREx with strict mode 0 because the fraction of pseudo-reads with more than two errors is smaller. This is especially true for R1 where the error-rate of CAREx remains greater than Konnector2’s regardless of the selected strictness. Still, more restrictive extension leads to a decrease in error-rate, and reaches smaller values than Konnector2 on R2, R3, and R4. Results for the two human datasets R3 and R4 are very similar. Note that edits may also be caused from genomic variations, for example single nucleotide polymorphisms (SNPs), compared to the used reference genome. Thus, the actual number of wrong nucleotides, for all tools, may be lower. As with simulated reads, the results of GapFiller are not competitive. We did not attempt the extension of R3 with GapFiller.

De novo assembly was performed with SPAdes v3.13.1 [26] and MEGAHIT v1.2.9 [27]. For each assembly we passed both connected reads and remaining unconnected reads to the software. Assembled contigs were analysed using QUAST v5.0.2 [28]. GapFiller was excluded from this analysis because of the previously reported sub-optimal results. Both Konnector2 and CAREx were run with disabled outward extension. We were unable to generate assemblies for R3 and R4 because of resource limitations.

**Table 5:** A selection of assembly metrics reported by QUAST for real datasets R1 and R2 assembled with SPAdes

	Unprocessed	CAREx			Konnector2	
		Strict 0	Strict 1	Strict 2	Standard	Preserve
*R1*						
Contigs $\ge$ 50,000 bp	540	670	629	650	633	642
N50	40,264	96,480	114,381	107,806	124,966	115,631
Misassembled contigs	672	626	608	638	639	648
*R2*						
Contigs $\ge$ 50,000 bp	25	44	28	23	16	14
N50	16,287	17,903	16,500	15,384	13,703	13,686
Misassembled contigs	200	195	293	327	512	474

**Table 6:** A selection of assembly metrics reported by QUAST for real datasets R1 and R2 assembled with MEGAHIT

	Unprocessed	CAREx			Konnector2	
		Strict 0	Strict 1	Strict 2	Standard	Preserve
*R1*						
Contigs $\ge$ 50,000 bp	660	620	695	703	656	667
N50	58,667	50,952	63,487	76,406	66,296	65,654
Misassembled contigs	1,235	1,463	1,437	1,411	1,379	1,429
*R2*						
Contigs $\ge$ 50,000 bp	124	111	138	139	148	136
N50	29,625	29,676	33,338	32,784	33,744	33,669
Misassembled contigs	243	332	263	259	230	240

Excerpts of the assembly reports generated by QUAST are presented in Table 5 for SPAdes and in Table 6 for MEGAHIT. Full reports are provided in Additional file 1: Section C.

On dataset R1 with SPAdes, both CAREx and Konnector2 are able to improve the N50 value and the number of misassembled contigs compared to an assembly with unprocessed reads. Konnector2 achieves the greatest N50 value whereas CAREx produces the lowest number of misassemblies. On dataset R2 with SPAdes only CAREx is able to achieve a better assembly than with unprocessed reads.

For assemblies produced by MEGAHIT, read extension leads to improved results for both R1 and R2 with extended reads from both Konnector2 and CAREx. In direct comparison of the extension tools, CAREx produced the best results on R1 whereas Konnector produced the best results on R2. This is in contrast to the SPAdes assemblies where the order is reversed.

Overall, read extension prior to genome assembly can be beneficial. For CAREx, the strictness of extension affects the assembly but judging from the presented results no level of strictness immediately outperforms the others. A lower strictness may produce more errors in the generated pseudo-long reads but at the same time will also produce a greater total number of pseudo-long reads with few or zero errors. Similarly, the benefits of disabling the integrated error-correction of Konnector2 depend on the dataset and on the used assembler.

### Runtime and memory consumption

**Table 7:** Total program runtime and CPU (GPU) peak memory consumption in GB for the datasets S2, S3, S4, and R3

S2	Threads	Runtime [minutes]	Memory [GB]
CAREx (CPU)	64	43	21
CAREx (GPU, CPU tables)	2+20	8	21 (8)
CAREx (GPU, GPU tables)	2	6	7 (37)
GapFiller	1	3855	4
Konnector2	64	24	11

S3	Threads	Runtime [minutes]	Memory [GB]
CAREx (CPU)	64	99	21
CAREx (GPU, CPU tables)	2+20	19	21 (8)
CAREx (GPU, GPU tables)	2	14	7 (37)
Konnector2	64	62	11

S4	Threads	Runtime [minutes]	Memory [GB]
CAREx (CPU)	64	365	42
CAREx (GPU, CPU tables)	2+20	44	40 (14)
CAREx (GPU, GPU tables)	2	38	14 (72)
Konnector2	64	126	11

R3	Threads	Runtime [hours:minutes]	Memory [GB]
CAREx (CPU)	64	23:18	196
CAREx (GPU, CPU tables)	2+20	3:20	187 (63)
Konnector2	64	32:27	201
Konnector2 (preserve)	64	27:43	201

Benchmarks were conducted on a single-socket Linux workstation comprising an AMD EPYC 7713P 64-Core processor, 512 GB DDR4 RAM, and an NVIDIA A100 PCIe GPU with 80 GB HBM2e memory. CUDA Toolkit 11.8 was used. Total program runtime and peak memory consumption for read extension of datasets S2, S3, S4, and R3 are presented in Table 7, showing the scaling of runtime with different insert sizes and number of reads. CAREx was run with a memory limit of 200 GB. CAREx GPU with CPU tables used 20 threads for hashing on the CPU and 2 threads for read extension on the GPU.

On the simulated datasets Konnector2 is the fastest of the CPU-based tools. CAREx (CPU) is up to three times slower. GapFiller does not provide an option for multi-threading which results in a much longer processing time compared to Konnector2 and CAREx (CPU). On real-world dataset R3, CAREx (CPU) is faster than Konnector2.

The GPU-accelerated versions of CAREx are significantly faster than the CPU-based tools. Our GPU-based implementation is around seven times faster than our CPU version, allowing for the processing of 600 M human reads of length 148 in a few hours instead of a day.

Our peak memory usage is reached during hash table construction, when all key-value pairs of all tables are materialized before compacting them into buckets. If the memory limit prohibits the construction of all tables at once, tables are created in batches to reduce the memory consumption which in turn leads to slightly increased construction times. For example, if a memory limit of 480 GB is set for R3, construction times decreases by 9 min and 1 min for (CPU) and (GPU, CPU tables), respectively. However, these gains in runtime are negligible compared to the total runtime. The peak memory usage in this case is around 380 GB.

## Conclusions

In recent years, third-generation sequencing technologies have emerged, which can provide longer reads. Nevertheless, NGS is still widely used today because of high throughput and low error rates. In addition, PE sequencing strategies are often used, where pairs of short reads are produced from two ends of a DNA fragment. However, producing pseudo-long reads from PE short read data can be challenging when insert sizes exceed twice the read length. CAREx addresses this task by iterative MSA construction. It is able to accurately connect a large fraction of read pairs and exceeds the total number of pseudo-long reads produced by other tools.

To tackle the inevitable computational overhead of computing large amounts of MSAs CAREx can take advantage of both multiple CPU threads, and GPUs, for faster processing. Our GPU-accelerated version is up to 8 times faster than the fastest CPU tool (Konnector2). However, we require many hash tables for best results which in turn increases memory consumption. Users of CAREx can therefore set the number of hash tables to less than 48 to reduce memory usage on platforms with limited amount of RAM which may decrease the number of connected read pairs.

As seen from the results, when a dataset has a large variation in insert size a large fraction of edits is caused by incorrect pseudo-read lengths. To further improve CAREx, we would need to identify those cases during computation, and either handle them differently, or discard their extension entirely. One possible approach to identification would be to first extend all read pairs to maximum insert size, and then determine whether there are multiple possible positions where the mate could be placed. However, this approach could significantly increase the runtime.

In the future we may extend our approach to metagenomic samples. This would require additional preprocessing to separate input reads by species. Another topic of interest is the applicability of our approach to long-read sequencing platforms. As we have shown, using MSAs can produce accurate extensions of reads. We believe that this method can also be adapted to long reads by using a pair-wise alignment method that can handle insertions and deletions, such as a semi-global alignment. Another interesting research direction is the application of machine learning methods (such deep neural networks) for making extension decisions based on constructed MSAs. Similar approaches have recently been demonstrated to improve the accuracy of variant calling [29].

### Supplementary Information

**Additional file 1.**

## Data Availability

Instructions to generated simulated datasets are given in Additional file 1. Dataset S1 can be downloaded from: (https://zenodo.org/doi/10.5281/zenodo.10378907). Real-world datasets are publicly available. R1: Accession number SRR988075. (https://trace.ncbi.nlm.nih.gov/Traces/?view=run_browser &acc=SRR988075). R2: Was used by [30]. (https://cloudstor.aarnet.edu.au/plus/s/f0f6cb1385704ae8403dfbf86dd622d8/download?path=%2F &files=Human_NA19240.7z). R3: Accession numbers SRR2052337, SRR2052338, SRR2052339, SRR2052342, SRR2052348, SRR2052352, and SRR2052354. (https://www.ebi.ac.uk/ena/browser/view/SRR2052337). R4: Accession numbers SRR1766553, SRR1766558, SRR1766560, SRR1766562, SRR1766581, SRR1766585, and SRR1766588. (https://www.ebi.ac.uk/ena/browser/view/SRR1766553). Project name: CAREx. Project home page: https://github.com/fkallen/CAREx. Operating system(s): Linux. Programming language: C++, CUDA. Other requirements: The GPU version requires CUDA toolkit $\ge$ 11. License: GPLv3. Any restrictions to use by non-academics: No additional restrictions.
