# Supplementary material for: CAREx: context-aware read extension of paired-end sequencing data
Source: BMC Bioinformatics. 2024 May 10;25:186. doi: 10.1186/s12859-024-05802-w (PMC11088031; doi:10.1186/s12859-024-05802-w)
Supplement: Supplementary file 1 — Additional file 1. [file 12859_2024_5802_MOESM1_ESM.pdf]

CAREx: Context-Aware Read Extension from Paired-End Sequencing Data

Additional File 1: Supplementary information

Table of Contents

A Command lines....2
S1....2
S2,S5,S6....3
S3....4
S4....5
S7....6
S8....7
R1....8
R2....9
R3, R4....10
De novo assembly....11
Generating simulated datasets....11
Evaluation....11
B Simulated results....12
Datasets....12
Number of connected read pairs....12
Number of error-free connections....13
Number of connections with less than three errors....13
Total error-rate in outward extension....14
Pseudo-read lengths with outward extension....14
C Real-world results....15
Datasets....15
R1 Gap statistics....15
R2 Gap statistics....16
R3 Gap statistics....16
R4 Gap statistics....17
QUAST assembly reports - SPAdes....18
R1....18
R2....21
QUAST assembly reports - MEGAHIT....23
R1....23
R2....26

A Command lines

S1

GapFiller:

- GapFiller --output-prefix outputdir/ --seed1 input_1.fastq --seed2 input_2.fastq --seed-ins 300 --seed-var 5

Konnector2

- konnector -j 64 -k 32 -f 280 -F 320 -b 10G -o outputfileprefix input_1.fastq input_2.fastq

CAREx (CPU)

- carex-cpu -i input_1.fastq -i input_2.fastq -c 30 -d . -q --excludeAmbiguous -m 200G -t 64 --minFragmentSize 280 --maxFragmentSize 320 --eo extended.fastq --ro remaining.fastq --outputRemaining --strictExtensionMode 0

CAREx (GPU, CPU tables)

- carex-gpu -i input_1.fastq -i input_2.fastq -c 30 -d . -q --excludeAmbiguous -m 200G -t 64 --minFragmentSize 280 --maxFragmentSize 320 --eo extended.fastq --ro remaining.fastq --outputRemaining --strictExtensionMode 0 -g 0 --warpcore 0 --gpuExtenderThreadConfig 2:20 --batchsize 8192

CAREx (GPU, GPU tables)

- carex-gpu -i input_1.fastq -i input_2.fastq -c 30 -d . -q --excludeAmbiguous -m 200G -t 64 --minFragmentSize 280 --maxFragmentSize 320 --eo extended.fastq --ro remaining.fastq --outputRemaining --strictExtensionMode 0 -g 0 --warpcore 1 --gpuExtenderThreadConfig 2:0 --batchsize 8192

S2,S5,S6

GapFiller:

- GapFiller --output-prefix outputdir/ --seed1 input_1.fastq --seed2 input_2.fastq --seed-ins 500 --seed-var 10

Konnector2

- konnector -j 64 -k 32 -f 460 -F 540 -b 10G -o outputfileprefix input_1.fastq input_2.fastq

CAREx (CPU)

- carex-cpu -i input_1.fastq -i input_2.fastq -c 30 -d . -q --excludeAmbiguous -m 200G -t 64 --minFragmentSize 460 --maxFragmentSize 540 --eo extended.fastq --ro remaining.fastq --outputRemaining --strictExtensionMode 0

CAREx (GPU, CPU tables)

- carex-gpu -i input_1.fastq -i input_2.fastq -c 30 -d . -q --excludeAmbiguous -m 200G -t 64 --minFragmentSize 460 --maxFragmentSize 540 --eo extended.fastq --ro remaining.fastq --outputRemaining --strictExtensionMode 0 -g 0 --warpcore 0 --gpuExtenderThreadConfig 2:20 --batchsize 8192

CAREx (GPU, GPU tables)

- carex-gpu -i input_1.fastq -i input_2.fastq -c 30 -d . -q --excludeAmbiguous -m 200G -t 64 --minFragmentSize 460 --maxFragmentSize 540 --eo extended.fastq --ro remaining.fastq --outputRemaining --strictExtensionMode 0 -g 0 --warpcore 1 --gpuExtenderThreadConfig 2:0 --batchsize 8192

S3

GapFiller:

- GapFiller --output-prefix outputdir/ --seed1 input_1.fastq --seed2 input_2.fastq --seed-ins 1000 --seed-var 30

Konnector2

- konnector -j 64 -k 32 -f 880 -F 1120 -b 10G -o outputfileprefix input_1.fastq input_2.fastq

CAREx (CPU)

- carex-cpu -i input_1.fastq -i input_2.fastq -c 30 -d . -q --excludeAmbiguous -m 200G -t 64 --minFragmentSize 880 --maxFragmentSize 1120 --eo extended.fastq --ro remaining.fastq --outputRemaining --strictExtensionMode 0

CAREx (GPU, CPU tables)

- carex-gpu -i input_1.fastq -i input_2.fastq -c 30 -d . -q --excludeAmbiguous -m 200G -t 64 --minFragmentSize 880 --maxFragmentSize 1120 --eo extended.fastq --ro remaining.fastq --outputRemaining --strictExtensionMode 0 -g 0 --warpcore 0 --gpuExtenderThreadConfig 2:20 --batchsize 8192

CAREx (GPU, GPU tables)

- carex-gpu -i input_1.fastq -i input_2.fastq -c 30 -d . -q --excludeAmbiguous -m 200G -t 64 --minFragmentSize 880 --maxFragmentSize 1120 --eo extended.fastq --ro remaining.fastq --outputRemaining --strictExtensionMode 0 -g 0 --warpcore 1 --gpuExtenderThreadConfig 2:0 --batchsize 8192

S4

GapFiller:

- GapFiller --output-prefix outputdir/ --seed1 input_1.fastq --seed2 input_2.fastq --seed-ins 1000 --seed-var 30

Konnector2

- konnector -j 64 -k 32 -f 880 -F 1120 -b 10G -o outputfileprefix input_1.fastq input_2.fastq

CAREx (CPU)

- carex-cpu -i input_1.fastq -i input_2.fastq -c 60 -d . -q --excludeAmbiguous -m 200G -t 64 --minFragmentSize 880 --maxFragmentSize 1120 --eo extended.fastq --ro remaining.fastq --outputRemaining --strictExtensionMode 0

CAREx (GPU, CPU tables)

- carex-gpu -i input_1.fastq -i input_2.fastq -c 60 -d . -q --excludeAmbiguous -m 200G -t 64 --minFragmentSize 880 --maxFragmentSize 1120 --eo extended.fastq --ro remaining.fastq --outputRemaining --strictExtensionMode 0 -g 0 --warpcore 0 --gpuExtenderThreadConfig 2:20 --batchsize 8192

CAREx (GPU, GPU tables)

- carex-gpu -i input_1.fastq -i input_2.fastq -c 60 -d . -q --excludeAmbiguous -m 200G -t 64 --minFragmentSize 880 --maxFragmentSize 1120 --eo extended.fastq --ro remaining.fastq --outputRemaining --strictExtensionMode 0 -g 0 --warpcore 1 --gpuExtenderThreadConfig 2:0 --batchsize 8192

S7

GapFiller:

- GapFiller --output-prefix outputdir/ --seed1 input_1.fastq --seed2 input_2.fastq --seed-ins 500 --seed-var 150

Konnector2

- konnector -j 64 -k 32 -f 300 -F 1100 -b 10G -o outputfileprefix input_1.fastq input_2.fastq

CAREx (CPU)

- carex-cpu -i input_1.fastq -i input_2.fastq -c 30 -d . -q --excludeAmbiguous -m 200G -t 64 --minFragmentSize 300 --maxFragmentSize 1100 --eo extended.fastq --ro remaining.fastq --outputRemaining --strictExtensionMode 0

CAREx (GPU, CPU tables)

- carex-gpu -i input_1.fastq -i input_2.fastq -c 30 -d . -q --excludeAmbiguous -m 200G -t 64 --minFragmentSize 300 --maxFragmentSize 1100 --eo extended.fastq --ro remaining.fastq --outputRemaining --strictExtensionMode 0 -g 0 --warpcore 0 --gpuExtenderThreadConfig 2:20 --batchsize 8192

CAREx (GPU, GPU tables)

- carex-gpu -i input_1.fastq -i input_2.fastq -c 30 -d . -q --excludeAmbiguous -m 200G -t 64 --minFragmentSize 300 --maxFragmentSize 1100 --eo extended.fastq --ro remaining.fastq --outputRemaining --strictExtensionMode 0 -g 0 --warpcore 1 --gpuExtenderThreadConfig 2:0 --batchsize 8192

S8

Note: All reads in S8 were reverse-complemented before extension to produce inward-oriented reads.

GapFiller:

- GapFiller --output-prefix outputdir/ --seed1 input_1.fastq --seed2 input_2.fastq --seed-ins 2500 --seed-var 100

Konnector2

- konnector -j 64 -k 32 -f 2100 -F 2900 -b 10G -o outputfileprefix input_1.fastq input_2.fastq

CAREx (CPU)

- carex-cpu -i input_1.fastq -i input_2.fastq -c 30 -d . -q --excludeAmbiguous -m 200G -t 64 --minFragmentSize 2100 --maxFragmentSize 2900 --eo extended.fastq --ro remaining.fastq --outputRemaining --strictExtensionMode 0

CAREx (GPU, CPU tables)

- carex-gpu -i input_1.fastq -i input_2.fastq -c 30 -d . -q --excludeAmbiguous -m 200G -t 64 --minFragmentSize 2100 --maxFragmentSize 2900 --eo extended.fastq --ro remaining.fastq --outputRemaining --strictExtensionMode 0 -g 0 --warpcore 0 --gpuExtenderThreadConfig 2:20 --batchsize 8192

R1

GapFiller:

- GapFiller --output-prefix outputdir/ --seed1 input_1.fastq --seed2 input_2.fastq --seed-ins 598 --seed-var 39

Konnector2

- konnector -j 64 -k 32 -f 442 -F 754 -b 15G --fastq --corrected-qual 32 -o outputfileprefix input_1.fastq input_2.fastq

Konnector2 (preserve)

- konnector -j 64 -k 32 -f 442 -F 754 -b 15G --preserve-reads --fastq --corrected-qual 32 -o outputfileprefix input_1.fastq input_2.fastq

CAREx (CPU)

- carex-cpu -i input_1.fastq -i input_2.fastq -c 30 -d . -q --excludeAmbiguous -m 200G -t 64 --minFragmentSize 442 --maxFragmentSize 754 --eo extended.fastq --ro remaining.fastq --outputRemaining --strictExtensionMode 0

CAREx (GPU, CPU tables)

- carex-gpu -i input_1.fastq -i input_2.fastq -c 30 -d . -q --excludeAmbiguous -m 200G -t 64 --minFragmentSize 442 --maxFragmentSize 754 --eo extended.fastq --ro remaining.fastq --outputRemaining --strictExtensionMode 0 -g 0 --warpcore 0 --gpuExtenderThreadConfig 2:20 --batchsize 8192

CAREx (GPU, GPU tables)

- carex-gpu -i input_1.fastq -i input_2.fastq -c 30 -d . -q --excludeAmbiguous -m 200G -t 64 --minFragmentSize 442 --maxFragmentSize 754 --eo extended.fastq --ro remaining.fastq --outputRemaining --strictExtensionMode 0 -g 0 --warpcore 1 --gpuExtenderThreadConfig 2:0 --batchsize 8192

R2

GapFiller:

- GapFiller --output-prefix outputdir/ --seed1 input_1.fastq --seed2 input_2.fastq --seed-ins 312 --seed-var 14

Konnector2

- konnector -j 64 -k 32 -f 256 -F 368 -b 3G --fastq --corrected-qual 32 -o outputfileprefix input_1.fastq input_2.fastq

Konnector2 (preserve)

- konnector -j 64 -k 32 -f 256 -F 368 -b 3G --preserve-reads --fastq --corrected-qual 32 -o outputfileprefix input_1.fastq input_2.fastq

CAREx (CPU)

- carex-cpu -i input_1.fastq -i input_2.fastq -c 30 -d . -q --excludeAmbiguous -m 200G -t 64 --minFragmentSize 256 --maxFragmentSize 368 --eo extended.fastq --ro remaining.fastq --outputRemaining --strictExtensionMode 0

CAREx (GPU, CPU tables)

- carex-gpu -i input_1.fastq -i input_2.fastq -c 30 -d . -q --excludeAmbiguous -m 200G -t 64 --minFragmentSize 256 --maxFragmentSize 368 --eo extended.fastq --ro remaining.fastq --outputRemaining --strictExtensionMode 0 -g 0 --warpcore 0 --gpuExtenderThreadConfig 2:20 --batchsize 8192

CAREx (GPU, GPU tables)

- carex-gpu -i input_1.fastq -i input_2.fastq -c 30 -d . -q --excludeAmbiguous -m 200G -t 64 --minFragmentSize 256 --maxFragmentSize 368 --eo extended.fastq --ro remaining.fastq --outputRemaining --strictExtensionMode 0 -g 0 --warpcore 1 --gpuExtenderThreadConfig 2:0 --batchsize 8192

R3, R4

Konnector2

- konnector -j 64 -k 32 -f 300 -F 1100 -b 200G --fastq --corrected-qual 32 -o outputfileprefix input_1.fastq input_2.fastq

Konnector2 (preserve)

- konnector -j 64 -k 32 -f 300 -F 1100 -b 200G --preserve-reads --fastq --corrected-qual 32 -o outputfileprefix input_1.fastq input_2.fastq

CAREx (CPU)

- carex-cpu -i input_1.fastq -i input_2.fastq -c 30 -d . -q --excludeAmbiguous -m 200G -t 64 --minFragmentSize 300 --maxFragmentSize 1100 --eo extended.fastq --ro remaining.fastq --outputRemaining --strictExtensionMode 0 --qualityScoreBits 2

CAREx (GPU, CPU tables)

- carex-gpu -i input_1.fastq -i input_2.fastq -c 30 -d . -q --excludeAmbiguous -m 200G -t 64 --minFragmentSize 300 --maxFragmentSize 1100 --eo extended.fastq --ro remaining.fastq --outputRemaining --strictExtensionMode 0 -g 0 --warpcore 0 --gpuExtenderThreadConfig 2:20 --batchsize 8192 --qualityScoreBits 2

De novo assembly

SPAdes

- spades.py -t 64 -m 240 --12 remainingreads --merged connectedreads -o spadesout

MEGAHIT

- ./megahit --12 remainingreads -r connectedreads -o megahitout

QUAST

- quast.py contigs.fasta -R genome.fasta -o quastout --plots-format pdf --labels “program name” --threads 64

Generating simulated datasets

We used the ART simulator to generate simulated datasets.
https://www.niehs.nih.gov/research/resources/software/biostatistics/art/index.cfm

- art_illumina -ss HS20 -ef -na -i genome.fa -p -l 100 -f 30 -m 300 -s 5 -o outputprefix
- art_illumina -ss HS20 -ef -na -i genome.fa -p -l 100 -f 30 -m 500 -s 10 -o outputprefix
- art_illumina -ss HS20 -ef -na -i genome.fa -p -l 100 -f 30 -m 1000 -s 30 -o outputprefix
- art_illumina -ss HS20 -ef -na -i genome.fa -p -l 100 -f 60 -m 1000 -s 30 -o outputprefix
- art_illumina -ss HSXt -ef -na -i genome.fa -p -l 150 -f 30 -m 500 -s 150 -o outputprefix
- art_illumina -ss HSXt -ef -na -i genome.fa -mp -l 150 -f 30 -m 2500 -s 100 -o outputprefix

Evaluation

Our custom evaluation scripts with usage examples can be found online under:
https://github.com/fkallen/CAREx/tree/master/evaluation
Our evaluation scripts for simulated data are tailored for ART simulated reads

B Simulated results

Datasets

Name	Organism	Coverage	Read pairs	Read length	Insert size	Stdev
S1	C. elegans	30x	15,042,855	100	300	5
S2	C. elegans	30x	15,042,855	100	500	10
S3	C. elegans	30x	15,042,855	100	1000	30
S4	C. elegans	60x	30,085,710	100	1,000	30
S5	D. melanogaster	30x	18,043,082	100	500	10
S6	Hum. Chr. 14	30x	13,243,307	100	500	10
S7	C. elegans	30x	10,114,534	150	500	150
S8	C. elegans	30x	10,028,550	150	2,500	100

Dataset S1 is available for download at https://zenodo.org/doi/10.5281/zenodo.10378907

Number of connected read pairs

	CAREx			GapFiller	Konnector2
	Strict 0	Strict 1	Strict 2		
S1	14,812,043	13,902,853	13,739,890	970,903	12,742,048
S2	14,651,002	13,533,838	13,355,908	1,247,502	11,358,161
S3	14,348,702	12,860,565	12,599,425		8,804,628
S4	28,884,561	26,566,406	26,268,679		7,681,882
S5	17,489,126	16,564,464	16,466,903	1,591,823	15,458,577
S6	12,767,428	11,581,453	11,443,877		8,295,535
S7	10,016,848	9,558,861	9,487,590		8,185,781
S8	9,436,488	8,384,118	8,266,592		4,118,248

Number of error-free connections

	CAREx			GapFiller	Konnector2
	Strict 0	Strict 1	Strict 2		
S1	14,564,100	13,826,113	13,697,492	855,812	11,881,879
S2	14,238,321	13,445,823	13,312,913	745,054	9,577,623
S3	13,765,667	12,786,049	12,569,592		5,956,933
S4	27,823,292	26,420,824	26,203,000		2,687,126
S5	17,243,989	16,487,140	16,415,467	960,089	13,110,655
S6	12,556,174	11,550,351	11,423,796		7,046,057
S7	9,695,451	9,488,850	9,442,647		7,595,997
S8	8,767,748	8,323,606	8,241,785		2,470,162

Number of connections with less than three errors

	CAREx			GapFiller	Konnector2
	Strict 0	Strict 1	Strict 2		
S1	14,726,164	13,887,307	13,732,906	939,891	12,733,910
S2	14,418,530	13,495,436	13,340,812	1,076,863	11,343,601
S3	13,989,075	12,827,375	12,590,193		8,767,093
S4	28,200,055	26,501,874	26,246,786		7,649,835
S5	17,380,217	16,537,889	16,452,493	1,381,925	15,445,237
S6	12,678,937	11,570,328	11,438,122		8,285,732
S7	9,820,018	9,521,260	9,465,198		8,159,663
S8	9,033,730	8,358,822	8,257,663		4,055,869

Total error-rate in outward extension

	CAREx		
	Strict 0	Strict 1	Strict 2
S1	0.00243	0.00167	0.00124
S2	0.00325	0.00176	0.00131
S3	0.00499	0.00285	0.00246
S4	0.00455	0.00250	0.00216
S5	0.00112	0.00062	0.00047
S6	0.00128	0.00085	0.00077
S7	0.00377	0.00221	0.00144
S8	0.00846	0.00524	0.00492

Pseudo-read lengths with outward extension

	CAREx strict 2		
	Min	Avg	Max
S1	285	607	760
S2	476	1,195	1,431
S3	889	2,639	3,184
S4	909	2,653	3,163
S5	468	1,201	1,399
S6	472	1,171	1,410
S7	302	1,049	2,672
S8	2,144	6,790	8,312

C Real-world results

Datasets

Name	Organism	Coverage	Read pairs	Read length	Insert size	Stdev
R1	D. melanogaster	64x	37,969,138	101	598	39
R2	Human Chr 21	33x	6,743,068	100	312	14
R3	Human (NA12878)	30x	304,615,512	148	546	117
R4	Human (NA24385)	31x	311,866,330	148	568	159

R1 Gap statistics

	CAREx			GapFiller	Konnector2	Konnector2 (preserve)
	Strict 0	Strict 1	Strict 2			
# connected	25,831,731	19,752,485	18,343,363	91,930	13,345,745	8,130,168
clipped alignment	2,237,433	1,424,927	1,203,212	9,032	507,329	316,830
# connected, not clipped	23,594,298	18,327,558	17,140,151	82,898	12,838,416	7,813,338
# edits	105,547,730	69,310,597	61,838,553	344,166	35,857,785	22,037,180
# bases	10,258,573,018	7,843,103,475	7,282,918,176	32,374,129	5,294,291,459	3,221,407,588
Error rate	0.0103	0.0088	0.0085	0.0106	0.0068	0.0068
# edit distance = 0	10,640,755	9,142,463	8,706,361	27,270	5,523,457	3,362,086
# edit distance <= 2	16,929,327	13,742,782	12,953,880	50,391	10,258,025	6,246,026

R2 Gap statistics

	CAREx			GapFiller	Konnector2	Konnector2 (preserve)
	Strict 0	Strict 1	Strict 2			
# connected	6,078,379	5,218,681	4,979,782	480,295	4,959,009	4,071,912
clipped alignment	40,512	26,745	21,694	11,870	19,789	14,937
# connected, not clipped	6,037,867	5,191,936	4,958,088	468,425	4,939,220	4,056,975
# edits	1975429	1,148,156	996,224	262,164	1,310,462	1,070,233
# bases	683,415,397	586,714,486	559,939,801	49,155,137	557,276,981	458,251,262
Error rate	0.0029	0.0020	0.0018	0.0053	0.0024	0.0023
# edit distance = 0	5,086,769	4,572,123	4,391,947	357,562	3,997,955	3,283,030
# edit distance <= 2	5,933,317	5,145,724	4,920,987	444,358	4,924,912	4,043,775

R3 Gap statistics

	CAREx			Konnector2	Konnector2 (preserve)
	Strict 0	Strict 1	Strict 2		
# connected	234,159,581	191,822,131	187,059,027	139,645,394	130,715,462
clipped alignment	2,956,034	1,367,922	1,229,124	723,964	565,611
# connected, not clipped	231,203,547	190,454,209	185,829,903	138,921,430	130,149,851
# edits	129,110,212	65,036,295	58,853,684	51,970,275	47,796,030
# bases	63,195,133,321	49,912,340,596	48,603,680,334	35,389,329,727	32,540,892,690
Error rate	0.00204	0.00130	0.00121	0.00147	0.00147
# edit distance = 0	181,269,284	159,542,446	156,547,109	107,547,655	101,020,580
# edit distance <= 2	224,361,216	187,467,454	183,265,508	137,778,101	129,017,669

R4 Gap statistics

	CAREx			Konnector2	Konnector2 (preserve)
	Strict 0	Strict 1	Strict 2		
# connected	244,470,826	198,099,919	193,174,725	143,336,871	133,201,714
clipped alignment	3,151,385	1,445,618	1,304,533	791,744	609,876
# connected, not clipped	241,319,441	196,654,301	191,870,192	142,545,127	132,591,838
# edits	145,107,893	72,489,003	65,662,750	57,131,424	52,301,066
# bases	69,945,838,613	54,903,786,786	53,476,441,950	38,853,129,373	35,549,349,321
Error rate	0.00207	0.00132	0.00123	0.00147	0.00147
# edit distance = 0	185,571,291	162,360,191	159,352,140	108,309,173	100,960,926
# edit distance <= 2	233,404,494	193,209,547	188,909,602	141,247,804	131,309,573

QUAST assembly reports - SPAdes

R1

	Unprocessed	CAREx			Konnector2	Konnector2 (preserve)
		Strict 0	Strict 1	Strict 2		
# contigs (>= 0 bp)	168,601	47,731	34,755	30,178	25,902	35,192
# contigs (>= 1000 bp)	7,450	4,534	4,221	4,202	3,595	3,784
# contigs (>= 5000 bp)	3,993	2,291	2,187	2,219	2,006	2,107
# contigs (>= 10000 bp)	2,740	1,737	1,629	1,677	1,499	1,608
# contigs (>= 25000 bp)	1,322	1,127	1,051	1,076	1,000	1,041
# contigs (>= 50000 bp)	540	670	629	650	633	642
Total length (>= 0 bp)	135,776,888	122,861,992	121,313,017	120,663,512	119,027,587	119,796,829
Total length (>= 1000 bp)	118,695,900	117,732,355	117,854,656	117,806,157	117,012,440	116,981,283
Total length (>= 5000 bp)	110,263,424	112,602,210	113,070,359	113,168,573	113,236,504	112,978,431
Total length (>= 10000 bp)	101,238,920	108,633,906	109,096,233	109,334,796	109,545,950	109,316,964
Total length (>= 25000 bp)	78,215,180	98,653,468	99,636,728	99,581,052	101,336,120	100,057,626
Total length (>= 50000 bp)	50,768,465	82,199,440	84,538,940	84,126,768	87,976,837	85,589,264
# contigs	9,540	5,696	5,092	4,953	3,957	4,224
Largest contig	479,630	686,266	722,108	833,613	1,121,191	624,240
Total length	120,163,302	118,582,786	118,491,282	118,365,384	117,305,868	117,329,377
Reference	120,381,546	120,381,546	120,381,546	120,381,546	120,381,546	120,381,546

length						
GC (%)	42.42	42.48	42.49	42.5	42.51	42.51
Reference GC (%)	42.41	42.41	42.41	42.41	42.41	42.41
N50	40,264	96,480	114,381	107,806	124,966	115,631
NG50	40,227	93,654	111,475	104,048	118,398	112,555
N75	16,981	39,439	42,282	42,763	49,889	46,046
NG75	16,910	37,531	39,967	39,700	46,028	42,045
L50	750	337	286	302	261	279
LG50	752	346	295	311	274	292
L75	1,897	822	724	751	634	693
LG75	1,907	857	758	788	682	745
# misassemblies	811	843	868	902	938	915
# misassembled contigs	672	626	608	638	639	648
Misassembled contigs length	33,919,038	54,227,737	58,604,328	58,014,861	63,757,076	61,500,357
# local misassemblies	1,041	1,025	1,046	1,081	1,105	1,110
# scaffold gap ext. mis.	0	0	0	0	0	0
# scaffold gap loc. mis.	0	0	0	0	0	0
# unaligned mis. contigs	414	334	302	272	165	171
# unaligned contigs	2133 + 940 part	1368 + 881 part	1150 + 845 part	1075 + 815 part	920 + 617 part	941 + 634 part
Unaligned length	6,388,977	4,950,932	4,875,128	4,773,361	4,248,520	4,245,219
Genome fraction (%)	93.99	94.04	94.04	94.02	93.7	93.69
Duplication ratio	1.01	1.01	1	1	1	1
# N's per 100 kbp	0	0	0	0	0	0
# mismatches	509.64	511.03	511.81	511.98	514.39	514.07

per 100 kbp						
# indels per 100 kbp	105.98	106.79	106.74	106.71	109.4	109.27
Largest alignment	335,133	550,421	574,434	453,089	480,463	462,666
Total aligned length	113,344,639	113,281,572	113,273,651	113,242,567	112,736,407	112,751,289
NA50	33,683	66,403	73,603	71,375	78,870	73,662
NGA50	33,641	64,881	71,669	70,252	76,158	71,388
NA75	14,846	29,124	30,760	30,970	34,983	32,559
NGA75	14,734	27,801	29,337	29,241	31,573	29,741
LA50	926	490	447	462	418	440
LGA50	929	504	460	476	438	461
LA75	2,270	1,159	1,069	1,087	975	1,038
LGA75	2,281	1,207	1,116	1,137	1,044	1,111

R2

	Unprocessed	CAREx			Konnector2	Konnector2 (preserve)
		Strict 0	Strict 1	Strict 2		
# contigs (>= 0 bp)	14,789	9,694	8,261	8,007	6,720	7,448
# contigs (>= 1000 bp)	3,347	3,144	3,405	3,573	3,839	3,837
# contigs (>= 5000 bp)	1,928	1,804	1,874	1,928	2,032	2,022
# contigs (>= 10000 bp)	1,129	1,092	1,081	1,079	1,067	1,061
# contigs (>= 25000 bp)	274	315	290	269	219	229
# contigs (>= 50000 bp)	25	44	28	23	16	14
Total length (>= 0 bp)	34,200,720	33,569,442	33,391,299	33,298,477	32,924,672	33,018,856
Total length (>= 1000 bp)	32,546,620	32,559,839	32,535,460	32,480,878	32,307,803	32,324,800
Total length (>= 5000 bp)	28,909,530	29,071,635	28,517,823	28,215,808	27,581,263	27,540,615
Total length (>= 10000 bp)	23,149,272	23,911,149	22,784,874	22,084,405	20,678,683	20,607,140
Total length (>= 25000 bp)	9,768,880	11,940,062	10,536,101	9,544,018	7,675,973	7,906,093
Total length (>= 50000 bp)	1,535,544	2,783,745	1,793,807	1,469,867	961,632	865,678
# contigs	3,847	3,561	3,821	3,992	4,269	4,275
Largest contig	82,949	126,285	112,201	112,198	90,182	108,889
Total length	32,905,112	32,860,807	32,837,399	32,786,927	32,627,583	32,649,528
Reference length	40,988,574	40,988,574	40,988,574	40,988,574	40,988,574	40,988,574
GC (%)	40.71	40.7	40.69	40.68	40.68	40.67
Reference GC (%)	40.93	40.93	40.93	40.93	40.93	40.93
N50	16,287	17,903	16,500	15,384	13,703	13,686
NG50	12,348	13,327	12,045	11,592	10,114	10,056
N75	8,605	9,375	8,381	7,964	7,204	7,206
NG75	3,069	3,179	2,908	2,733	2,499	2,515
L50	607	531	584	624	692	696

LG50	891	795	872	931	1,049	1,050
L75	1,295	1,169	1,283	1,359	1,517	1,519
LG75	2,394	2,225	2,449	2,600	2,914	2,900
# misassemblies	209	208	318	350	580	526
# misassembled contigs	200	195	293	327	512	474
Misassembled contigs length	3,583,491	3,898,745	5,524,109	5,414,538	7,703,457	6,890,369
# local misassemblies	115	113	118	130	130	130
# scaffold gap ext. mis.	0	0	0	0	0	0
# scaffold gap loc. mis.	0	0	0	0	0	0
# unaligned mis. contigs	7	8	8	7	4	4
# unaligned contigs	23 + 13 part	23 + 10 part	19 + 13 part	18 + 13 part	14 + 10 part	12 + 11 part
Unaligned length	34,945	33,680	31,253	32,992	26,318	21,686
Genome fraction (%)	80.1	80.02	79.95	79.82	79.43	79.49
Duplication ratio	1	1	1	1	1	1
# N's per 100 kbp	0	0	0	0	0	0
# mismatches per 100 kbp	152.36	154.06	155.72	156.91	170.18	167.1
# indels per 100 kbp	34.45	34.54	34.59	34.49	36.17	35.7
Largest alignment	82,399	117,777	95,674	108,818	90,171	108,878
Total aligned length	32,834,507	32,795,307	32,774,721	32,717,725	32,557,144	32,583,321
NA50	15,466	16,719	15,129	14,473	11,946	12,180
NGA50	11,646	12,519	10,818	10,498	8,704	8,894
NA75	8,174	8,736	7,691	7,308	6,200	6,287
NGA75	2,812	2,929	2,666	2,488	2,165	2,187
LA50	636	565	644	678	794	783
LGA50	937	844	960	1,013	1,204	1,183
LA75	1,365	1,241	1,411	1,481	1,742	1,716
LGA75	2,545	2,376	2,694	2,859	3,372	3,304

QUAST assembly reports - MEGAHIT

R1

	Unprocessed	CAREx			Konnector2	Konnector2 (preserve)
		Strict 0	Strict 1	Strict 2		
# contigs (>= 0 bp)	31,158	55,994	38,680	32,139	35,035	35,670
# contigs (>= 1000 bp)	6,625	7,310	6,481	5,903	6,256	6,398
# contigs (>= 5000 bp)	3,172	3,419	2,996	2,687	2,916	3,003
# contigs (>= 10000 bp)	2,295	2,424	2,181	1,968	2,112	2,136
# contigs (>= 25000 bp)	1,266	1,308	1,259	1,220	1,244	1,259
# contigs (>= 50000 bp)	660	620	695	703	656	667
Total length (>= 0 bp)	130,551,140	138,474,437	134,116,242	132,466,859	133,149,736	133,383,281
Total length (>= 1000 bp)	120,908,702	121,608,576	122,139,143	122,321,265	121,923,972	121,973,445
Total length (>= 5000 bp)	113,437,247	113,007,963	114,750,500	115,414,234	114,718,718	114,611,890
Total length (>= 10000 bp)	107,224,694	105,886,443	109,003,129	110,343,414	109,065,466	108,432,473
Total length (>= 25000 bp)	90,502,812	87,858,774	94,025,460	98,179,114	94,978,386	94,364,469
Total length (>= 50000 bp)	68,744,299	63,543,646	73,688,748	79,652,148	73,900,468	73,288,279
# contigs	10,705	12,680	10,978	9,923	10,628	10,847
Largest contig	579,907	541,436	650,302	714,351	588,383	617,864
Total length	123,648,769	125,125,781	125,099,943	124,982,669	124,818,786	124,924,311
Reference	120,381,546	120,381,546	120,381,546	120,381,546	120,381,546	120,381,546

length						
GC (%)	42.34	42.31	42.32	42.32	42.3	42.3
Reference GC (%)	42.41	42.41	42.41	42.41	42.41	42.41
N50	58,667	50,952	63,487	76,406	66,296	65,654
NG50	60,108	53,498	66,923	81,556	70,353	69,304
N75	22,847	20,034	25,308	30,520	26,411	25,759
NG75	25,322	22,725	29,426	35,313	30,034	29,461
L50	533	601	499	425	458	480
LG50	506	556	462	395	426	446
L75	1,360	1,577	1,252	1,062	1,191	1,233
LG75	1,258	1,410	1,123	956	1,074	1,109
# misassemblies	1,573	1,920	1,890	1,894	1,863	1,865
# misassembled contigs	1,235	1,463	1,437	1,411	1,379	1,429
Misassembled contigs length	45,641,470	44,505,887	50,910,503	56,627,307	52,114,864	51,994,693
# local misassemblies	1,634	1,940	1,750	1,774	1,791	1,818
# scaffold gap ext. mis.	0	0	0	0	0	0
# scaffold gap loc. mis.	0	0	0	0	0	0
# unaligned mis. contigs	906	1,108	1,016	940	956	915
# unaligned contigs	2142 + 1485 part	2289 + 1665 part	2195 + 1612 part	2148 + 1545 part	2177 + 1493 part	2198 + 1471 part
Unaligned length	6,961,251	7,460,396	7,592,068	7,614,157	7,387,432	7,412,965
Genome fraction (%)	95.08	95.17	95.27	95.32	95.26	95.28
Duplication ratio	1.02	1.03	1.03	1.02	1.03	1.03
# N's per 100 kbp	0	0	0	0	0	0
# mismatches	556.24	573.46	570.56	567.76	577.27	573.89

per 100 kbp						
# indels per 100 kbp	109.09	109.16	109.25	109.7	111.21	110.9
Largest alignment	436,518	394,185	462,272	450,834	462,783	414,382
Total aligned length	115,947,842	116,800,237	116,744,075	116,658,911	116,663,348	116,738,991
NA50	46,313	40,314	48,906	55,613	50,211	49,564
NGA50	47,688	43,280	51,957	58,697	53,056	52,623
NA75	18,860	16,261	20,196	23,338	20,460	19,857
NGA75	20,709	18,491	23,089	26,706	23,468	22,817
LA50	707	780	679	607	641	661
LGA50	672	724	632	567	598	617
LA75	1,746	1,992	1,662	1,460	1,600	1,639
LGA75	1,623	1,786	1,499	1,323	1,449	1,479

R2

	Unprocessed	CAREx			Konnector2	Konnector2 (preserve)
		Strict 0	Strict 1	Strict 2		
# contigs (>= 0 bp)	3,144	5,026	4,433	3,789	3,207	3,142
# contigs (>= 1000 bp)	2,055	2,060	1,906	1,848	1,855	1,861
# contigs (>= 5000 bp)	1,405	1,416	1,311	1,299	1,265	1,289
# contigs (>= 10000 bp)	1,015	1,051	985	987	969	985
# contigs (>= 25000 bp)	434	460	454	474	466	462
# contigs (>= 50000 bp)	124	111	138	139	148	136
Total length (>= 0 bp)	33,820,992	34,766,845	34,654,315	34,485,141	34,194,383	34,174,385
Total length (>= 1000 bp)	33,314,455	33,591,236	33,663,131	33,678,339	33,597,462	33,608,652
Total length (>= 5000 bp)	31,661,732	31,991,917	32,237,180	32,357,321	32,164,793	32,204,928
Total length (>= 10000 bp)	28,802,002	29,354,113	29,847,810	30,080,786	30,035,094	29,983,333
Total length (>= 25000 bp)	19,414,512	19,797,825	21,058,137	21,699,237	21,724,393	21,513,049
Total length (>= 50000 bp)	8,727,052	7,871,509	9,877,551	9,901,595	10,773,227	10,114,974
# contigs	2,445	2,632	2,441	2,354	2,259	2,243
Largest contig	187,558	150,517	214,235	218,521	166,898	230,132
Total length	33,589,431	33,982,001	34,030,855	34,027,297	33,879,115	33,871,774
Reference length	40,988,574	40,988,574	40,988,574	40,988,574	40,988,574	40,988,574
GC (%)	40.84	40.86	40.84	40.83	40.8	40.8
Reference GC (%)	40.93	40.93	40.93	40.93	40.93	40.93
N50	29,625	29,676	33,338	32,784	33,744	33,669
NG50	23,005	23,771	26,040	27,067	27,025	26,978
N75	15,406	15,660	17,815	18,293	18,429	17,679
NG75	6,819	7,375	8,327	8,541	8,443	8,408

L50	339	357	315	313	301	305
LG50	480	489	432	428	419	423
L75	727	747	664	653	637	647
LG75	1,248	1,212	1,084	1,058	1,047	1,068
# misassemblies	270	383	301	300	257	280
# misassembled contigs	243	332	263	259	230	240
Misassembled contigs length	7,354,137	8,861,690	8,846,643	7,836,179	7,460,938	8,256,397
# local misassemblies	161	185	165	181	177	172
# scaffold gap ext. mis.	0	0	0	0	0	0
# scaffold gap loc. mis.	0	0	0	0	0	0
# unaligned mis. contigs	8	24	17	14	14	13
# unaligned contigs	9 + 14 part	50 + 32 part	46 + 29 part	36 + 27 part	22 + 23 part	24 + 27 part
Unaligned length	22,831	69,783	66,810	59,198	47,048	52,363
Genome fraction (%)	81.44	81.91	82.07	82.06	81.9	81.88
Duplication ratio	1.01	1.01	1.01	1.01	1.01	1.01
# N's per 100 kbp	0	0	0	0	0	0
# mismatches per 100 kbp	178.9	204.92	197.26	196.42	192.76	187.81
# indels per 100 kbp	37.43	39.32	38.97	38.84	38.84	38.37
Largest alignment	157,650	143,017	214,040	218,470	166,865	226,321
Total aligned length	33,474,245	33,786,000	33,856,814	33,858,588	33,728,738	33,719,105
NA50	26,040	25,467	28,360	29,687	29,778	29,292
NGA50	20,260	20,248	22,616	23,459	24,212	23,265
NA75	13,821	13,575	14,684	15,541	16,236	15,394
NGA75	6,044	6,043	7,020	7,457	7,135	7,100
LA50	386	410	365	360	341	355
LGA50	547	564	503	491	473	491

LA75	829	864	779	751	721	750
LGA75	1,414	1,419	1,268	1,223	1,192	1,237
